# Performance Evaluation of COVID-19 Proximity Detection Using Bluetooth LE Signal

**DOI:** 10.1109/ACCESS.2021.3064323

**Published:** 2021-03-08

**Authors:** Zhuoran Su, Kaveh Pahlavan, Emmanuel Agu

**Affiliations:** 1 Center for Wireless Information Network StudiesElectrical and Computer Science DepartmentWorcester Polytechnic Institute 8718 Worcester MA 01609 USA; 2 Computer Science DepartmentWorcester Polytechnic Institute 8718 Worcester MA 01609 USA

**Keywords:** COVID-19, proximity detection, RSSI features, PACT, classical estimation theory, BLE, machine learning

## Abstract

The risk of COVID-19 transmission increases when an uninfected person is less than 6 ft from an infected person for longer than 15 minutes. Infectious disease experts working on the COVID-19 pandemic call this high-risk situation being Too Close for Too Long (TCTL). Consequently, the problem of detecting the TCTL situation in order to maintain appropriate social distance has attracted considerable attention recently. One of the most prominent TCTL detection ideas being explored involves utilizing the Bluetooth Low-Energy (BLE) Received Signal Strength Indicator (RSSI) to determine whether the owners of two smartphones are observing the acceptable social distance of 6 ft. However, using RSSI measurements to detect the TCTL situation is extremely challenging due to the significant signal variance caused by multipath fading in indoor radio channel, carrying the smartphone in different pockets or positions, and differences in smartphone manufacturer and type of the device. In this study we utilize the Mitre Range Angle Structured (MRAS) Private Automated Contact Tracing (PACT) dataset to extensively evaluate the effectiveness of Machine Learning (ML) algorithms in comparison to classical estimation theory techniques to solve the TCTL problem. We provide a comparative performance evaluation of proximity classification accuracy and the corresponding confidence levels using classical estimation theory and a variety of ML algorithms. As the classical estimation method utilizes RSSI characteristics models, it is faster to compute, is more explainable, and drives an analytical solution for the precision bounds proximity estimation. The ML algorithms, Support Vector Machines (SVM), Random Forest, and Gradient Boosted Machines (GBM) utilized thirteen spatial, time-domain, frequency-domain, and statistical features extracted from the BLE RSSI data to generate the same results as classical estimation algorithms. We show that ML algorithms can achieve 3.60%~19.98% better precision, getting closer to achievable bounds for estimation.

## I. Introduction

With the threat of COVID-19, a highly infectious virus, maintaining social distance is an effective way to prevent infection. Specifically, the risk of Covid-19 transmission increases when an uninfected person is less than 6 ft from an infected person for longer than 15 minutes (also called Too Close for Too Long (TCTL)). If the list of people who are TCTL to each smartphone user can be detected and tracked passively, they can be notified if the smartphone user tests positive for COVID-19. Existing opportunistic Radio Frequency (RF) positioning technologies can be used to track the infected smartphone user’s daily motion trajectory. The owners of neighboring smart devices can then be notified so that they can maintain social distance or get tested if they are in found to have been TCTL. Although the tradeoff between the benefits of COVID-19 mitigation using contact tracing and the intrusion on users’ privacy remains a difficult social political problem, scientific research in this area has recently gained momentum. With its short range and low energy consumption, the use of the ubiquitous Bluetooth Low Energy (BLE) signal has attracted significant attention. This led Massachusetts Institute of Technology (MIT), Boston, MA, to lead the Private Automated Contact Tracing (PACT) consortium [1] to make available several high quality BLE Received Signal Strength Indicator (RSSI) datasets, which were gathered in a variety of proximity scenarios. Their goal was to challenge research and development community to discover a solution to this timely and important problem. The reliability analysis of RSSI based BLE ranging is a complex problem because of the significant variance in the measured RSSI signal due to the complexity of the multipath indoor radio propagation causing extensive signal attenuations, fading and interference from other devices operating in unlicensed 2.4 GHz ISM bands. In prior work, real world measurements studies and characterization of proximity detection using BLE RSSI have been conducted in a variety of scenarios [2]. Some prior work has proposed approaches to improve RSSI-based proximity estimation by integrating data from other sensors including light [3], accelerometer and gyroscope [4], and user-sensed motion [5]. Some other authors have incorporated information on the place type [6], user context [7], sensed crowd [8], social context [5], [9], social circles [10], indoor-outdoor detection [11] and place co-location [12]. A modified path loss model has also been utilized [13]. Beyond proximity other authors used RSSI to estimate the mutual orientation between users [14] and the energy consumption of BLE RSSI proximity detection [15]. In this paper, we present the results of our extensive comparative performance evaluation of classical estimation theory and Machine Learning (ML) algorithms for social distance estimation using the BLE RSSI data. We utilized the MITRE Corporation Structured Angle dataset of the PACT project to share generate results and make our observations from this experiment. We begin by describing the MITRE Range Angle Structured (MRAS) PACT dataset followed by a review of RSSI features that are useful for distance estimation. Then, we present the classical estimation theory, which facilitates faster proximity computation in a more logically explainable manner, and ML algorithms that can be used to estimate user proximity with all the RSSI features. Finally, we provide our quantitative comparative performance evaluation of traditional and ML algorithms to solve the social distance estimation problem using the BLE signal. For the classical estimation theory results, we present the method for computing the confidence associated with the distance estimated using the BLE RSSI behavior models. We also derive bounds on the confidence of range estimation using the Cramer-Rao Lower Bound (CRLB). For the ML estimations, we classified thirteen spatial, time, frequency, and general statistical features of the BLE RSSI using three different algorithms: Support Vector Machine (SVM), Random Forest and Gradient Boosted Machines (GBM). The final outcome of this extensive study is the comparison of theoretical achievable bounds for social distance range estimation using BLE RSSI with the empirical results obtained using two theoretical RSSI behavior models and three ML classification algorithms. The RF cloud around wireless devices present an opportunity for designing novel cyberspace applications. The RF cloud contains features of the signal that reflect the multipath characteristics of the environment at each location. As a device moves, these multipath characteristics change rapidly opening an opportunity for other devices to observe these variations in characteristics and relate them to a location-dependent cyberspace application [16], [17]. The PACT project is a new opportunistic cyberspace application focused on an opportunistic proximity check application benefiting from the RF cloud of the BLE. The Center for Wireless Information Network Studies (CWINS) at the Worcester Polytechnic Institute (WPI), Worcester, MA has previous engagement in the PACT project and is now exploring systematic research in this field. Short term, the proximity detection BLE RSSI application can be investigated. Longer term research could involve extending the BLE signal by including other sensors. We build on our prior RSSI based positioning and motion and gesture detection research [18]–[20] for the current time. In future, we are planning to extend BLE by incorporating other opportunistic wireless signals including those from Wi-Fi and Ultra-wideband devices to increase the precision of range estimation.

## II. The PACT Promixity Datasets and Measurement Scenarios

**FIGURE 1.**
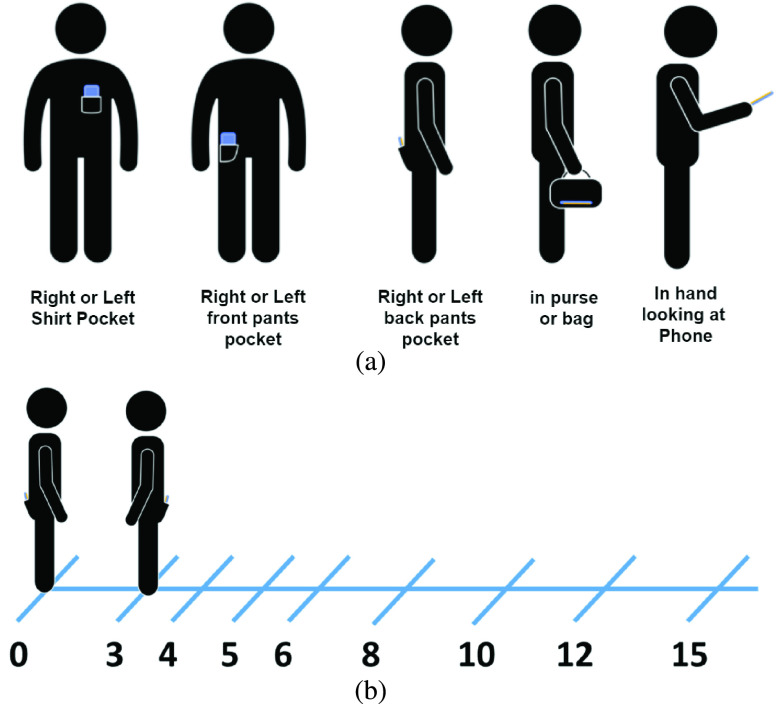
PACT measurement scenario for the MITRE Range Angle Structured dataset, (a) five scenarios for location of the smartphone, (b) eight distances for measurements of the RSSI data base. (Source: PACT website).

There are seven datasets made publicly available by the PACT consortium [1]. Compared with the other datasets, MRAS dataset is well documented. Moreover, it contains measurements in various testing scenarios at different distances, which are relevant to our study goals of comparing the performance of classical and ML algorithms using various features extracted from BLE RSSI measurements. The MRAS dataset also contains different environment and tester pose settings. Environment settings specify the properties of testing area, such as the room size and the tester’s location in the room. Tester settings defines the way devices are used by testers, in which way they hold the smartphones, and the poses of testers. Fig. 1 shows the location of device and 8 selected relative distances between testers for the MRAS database. Fig. 1.a shows the five scenarios emulating real life scenarios for position of the smartphone: in hand, in purse, in shirt pocket, in front pants pocket, and in the back pants pocket. Fig. 1.b shows the BLE RSSI measurement scenarios for short range of operation of up to 15 ft. The eight stationary locations for measurements begin at 3 ft, are increased at intervals, and end at 15 ft. The distances are identified with respect to a person who holds the smartphone with BLE beacons. The RSSI measurement data are collected by another person (a receiver) positioned at the eight labeled distances. In each test location identified in Fig. 1.b, 5–10 seconds measurements of BLE RSSI containing 300–400 samples of the RSSI are measured:

\begin{equation*}s(k)=\mathrm {RSSI}(t)\big |_{t=kT_{s}};\quad k=1,\ldots ,N ,\tag{1}\end{equation*}

where $N$ is the number of samples in a location and $T_{s}$ is the time interval between two adjacent RSSI measurement samples.

Table 1 summarizes various operation scenarios reported in the MRAS measurement database for collecting 300–400 samples of RSSI in each stationary dataset. The first two rows capture variations in the room size and locations of testers in the area. The detailed room size setting is not provided in the dataset, but a description of the scenarios is available. For example, the entrance to the bathroom of apartment is defined as small room, the kitchen is defined as medium room, and large living room is defined as large room [2]. The next two rows identify the types of the smartphone and the location of the smartphone on the tester body. The last row identifies testers pose that is either “sit” or “stand” at the marked location. These datasets were collected using three versions of Range-Angle Collection Protocol [1]: Short, Mid and Full. The Full protocol consists of 40 datasets with RSSI measurements at eight different distances shown in Fig. 1.b and we used these datasets for our performance evaluation for different proximity algorithms. We did not include the Short and Mid versions, which had only two different distances of 3 ft and 8 ft and did not offer adequate diversity in measurement distances.

**TABLE 1:** Scenarios for MITRE-Range-Angle-Structured Dataset

Scenarios	Settings
Description of the areas	Small Room, Medium Room, Large Room, Hallway, Outside
Location in the area	Center open, Center congested, Near wall open, Near wall congested
Type of Device	iPhone XR, iPhone 11, iPhone 8, iPhone6, iPhone 7, iPhone XS, iPhone 6 Plus
Device location on-body	In hand, In purse, Shirt pocket, Front pants pocket, Rear pants pocket
Tester Pose	Standing, Sitting

In our selected MRAS dataset, multipath fading characteristics and variation in the environment caused close to 30 dB difference in the values of RSSI in each measurement set and up to 30 dB variations in average RSSI in individual sets. The RSSI measurements in each location, defined by (1), are post-processed before feeding them into classical and ML algorithms respectively. In the classical estimation algorithms, the training data for RSSI behavior at each location (1), are averaged at each distance. The average RSSI at each location is then defined by:

\begin{equation*} {P_{r}}=\frac {1}{N}\sum \limits _{k=1}^{N}{s(k)}. \tag{2}\end{equation*}

This data post-processing for classical algorithms associates a single average RSSI measurement, $P_{r}$, to each location.

For ML techniques, the RSSI measurements at each location is grouped in overlapping windows of RSSI measurement vectors of length $L$, whose elements are defined by

\begin{align*} y(n)=\{s(k+n);k=0,1,\ldots L-1\};\quad n=1,\ldots ,N-L.\!\!\! \\ \tag{3}\end{align*}

This processing associates an $N-L$ set of $L$ dimensional vectors with each location. We utilized these post-processed RSSI data to perform our comparative performance evaluations for various classical and ML algorithms. Our performance criterion is the confidence in the decision made by the algorithm for the task of detecting the social distance of 6 ft using BLE RSSI measurements gathered using a smartphone at a given location.

## III. Features of RSSI Short Range Fading

Motion in the environment affects RF propagation in multipath indoor and urban areas and causes fading in the measured RSSI, which seriously challenges the precision of RSSI-based ranging [21]. The channel impulse response for two wireless devices communicating with a range $r$ in a multipath, indoor, or urban area with $N$-paths, is represented by [23]:

\begin{equation*}{h_{r}}({\alpha _{i}};{\tau _{i}};{\theta _{i}}) = \sum \limits _{i = 1}^{N} {\alpha _{i}} {e^{j{\theta _{i}}}}\delta (t - {\tau _{i}}),\end{equation*}

where ($\alpha _{i}$, $\tau _{i}$, $\theta _{i}$) are the magnitude, time of arrival, phase, and DOA of the i-th path.

\begin{align*} \mathrm {RSSI}(t)=&\sum \limits _{i = 1}^{N} {{{\left |{ {h_{r}({\alpha _{i}};{\tau _{i}};{\theta _{i}})} }\right |}^{2}}} \, = {\left |{ {\sum \limits _{i = 1}^{N} {\alpha _{i}} {e^{j{\theta _{i}}}}\delta (t - {\tau _{i}})} }\right |^{2}} \\=&{\left |{ {\sum \limits _{i = 1}^{N} {\alpha _{i}{e^{j{\theta _{i}}}}} } }\right |^{2}}\end{align*}

**FIGURE 2.**
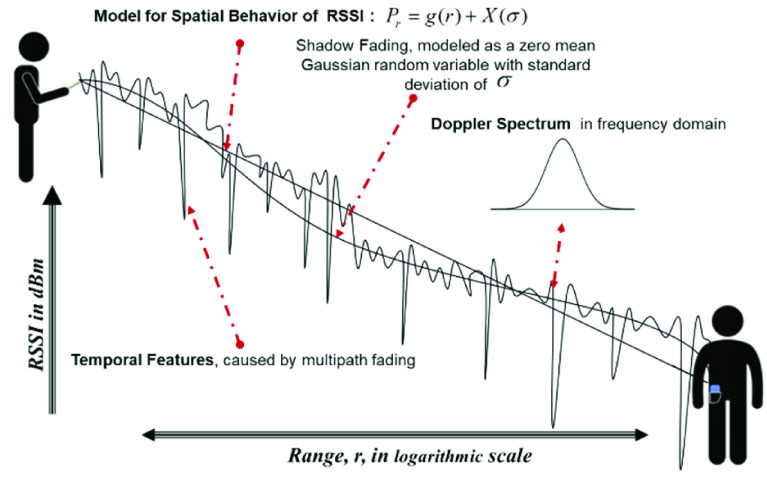
Variation of the received power in dB as a function of the logarithmic distance between the transmitter and the receiver and how we approach to model them for different purposes.

We can easily measure this RSSI from a transmitting wireless device without any synchronization with the source. Multipath arrival of the signal in indoor and urban areas, where the applications discussed in this paper operate, causes extensive fluctuations of the amplitude of the received signal in time. Fig. 2 illustrates the variation of the amplitude in dBm (RSSI) as a function of the logarithmic distance between the transmitter and the receiver, $r$, as a receiver moves away from a transmitter. This figure also shows how we approach the modeling of these variations of the RSSI for different applications. The instantaneous RSSI in a multipath environment always varies over time and with small local changes in distance or movement of objects located around the transmitter and the receiver antennas. The average of the RSSI decays as the distance increases and we use an RSSI model to predict the average received RSSI for calculating the coverage and interference of wireless networks and for RSSI-based cyberspace applications [23]. The distribution function of temporal changes in the signal is modeled with a few distribution functions to analyze the error rate of wireless modems. The Fourier Transform of these changes is referred to as the Doppler Spectrum, which reflects the speed of movement of objects or the device in the environment of operation. As the objects scattered in the area or the wireless devices move in the environment or we change the frequency of operation, characteristics of the multipath features fluctuate drastically and cause fading in measured RSSI. In the wireless communication literature, this phenomenon is discussed under temporal, frequency-selective, and spatial fading [22]. In this body of knowledge, RSSI features in space, time, and frequency are modelled using a few physical parameters that can be measured. These features can be utilized to improve the reliability of estimates generated by RSSI-base range estimation techniques.

### A. RSSI Spatial Features

In classical RSSI-base ranging we use the average RSSI in dBm for calculating the distance between an antenna and a device, $r$. The traditional method to model how the RSSI is related to the distance from the transmitter is to use linear regression and least square estimation to calculate the parameters of the model using empirical data [21]. The traditional statistical linear regression model for the spatial behavior of RSSI in dBm is:

\begin{equation*} \mathrm {RSSI}:{P_{r}} = {P_{0}} - 10\alpha {\log _{10}}(r) + X(\sigma ), \tag{4}\end{equation*}

in which $r$ is the distance, $X$ is a Gaussian random variable with variance $\sigma $, representing the shadow fading effects, $\alpha $ is the distance power gradient of the environment, and $P_{0}$ is the RSSI at a reference distance from the transmitter. Shadow fading represents variations of the RSSI from the linear regression line in dB caused by objects shadowing radio propagation paths between the transmitter and the receiver. We can use the traditional Least Square (LS) method of statistical modeling to estimate the RSSI spatial behavior model parameters, ($P_{0}$, $\alpha $, $\sigma $), using measured RSSI data in different scenarios provided by the PACT (section II) [21].

An alternative model for short range BLE RSSI is also reported in the literature [24], which we tested on the PACT database. Based on empirical measurements of BLE, this model suggests that the RSSI has an additional sinusoidal component:

\begin{align*} \mathrm {RSSI}:{P_{r}} \!=\! {P_{0}} - A\cos \,\left ({ {\frac {2\pi r}{\lambda }} }\right ) + 10\alpha \log (r)~\!+\! X(\sigma ). \\ \tag{5}\end{align*}

**FIGURE 3.**
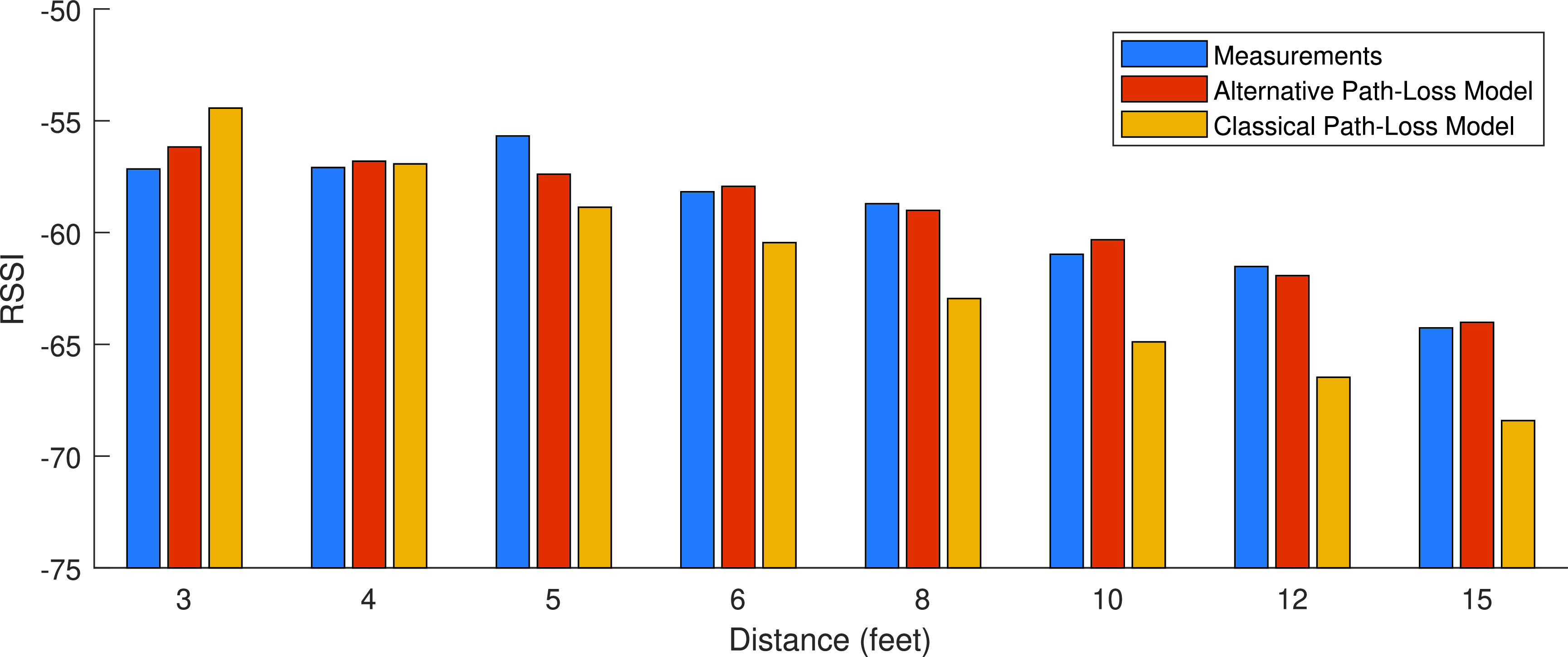
RSSI estimation using traditional RSSI behavior model and the alternative BLE specific model for a set of MRAS RSSI data.

Therefore, in addition to traditional model parameters, ($P_{0}$, $\alpha $, $\sigma $), this model has two new parameters ($A$, $\lambda $), $A$ is the amplitude scale of the sinusoidal part and $\lambda $ is its spatial wavelength, which we can also estimate using the LS algorithm. In this paper, we expand the effective range of this alternative BLE specific model to about 4.5 m (15 ft) and compare the results with produced using the traditional linear regression model described by (4). Fig. 3 shows the difference between classical linear regression RSSI model described by (4) and the alternative BLE-specific RSSI model described by (5) using a set of Mitre Corporation MRAS PACT data in eight distances. The BLE-specific RSSI model on the average provides a slightly better fit to data for predicting the measured RSSI values. In section V.A we compare the performance of classical range estimation algorithms when we used the MRAS RSSI database with both RSSI models.

### B. RSSI Features in Time Domain

In RSSI ranging, we measure a sequence of RSSI values in time at each location and estimate the range of these collective measurements. Because the person measuring the BLE RSSI at a location has slight body motions and the objects in the environment also move, the measured RSSI fluctuations in amplitude even when the transmitter and the receiver are held in specific locations. In the multipath RF propagation literature, these fluctuations are referred to as short range multipath fading and their characteristics are modelled for performance evaluation of wireless communications techniques [23]. By using a ML system designed for ranging, we can benefit from physical parameters of these fading models as features for training the algorithm. Traditionally in data science we use the mathematical statistics as features of these signals, the new features extracted from our understanding of the behavior of RF propagation in multipath environment can potentially improve the performance of the system. These features have been demonstrated to be very instrumental in RSSI-based gesture and motion detection [18], [19]. In this study, we evaluate the effectiveness of thirteen features of RSSI in estimating the social distancing between smartphone users utilizing BLE RSSI. Table 2 is a summary of the radio propagation and statistical features that we have selected to train the ML algorithms in our study. We calculated these features for all BLE RSSI measurements, $y(n)$, defined by (3) to form a vector that is used to train the ML algorithms in section IV.B. We have divide them into time-domain (section III.B), frequency domain (section III.C), and traditional statistical features (section III.D). The time domain RSSI features benefit from classical radio propagation modelling of these fluctuations, which includes fading rate, average fade duration, coherence time, and shape of distribution of fading, which we describe in the remaining subsection of this section.

**TABLE 2:** Summary of Thirteen Feature of the RSSI for Training Machine Learning Algorithms

Feature Name	Formula	Description
Time Domain Features		
Fade Duration	\begin{equation*} \tau (\rho ) = \frac {{{e^{\rho ^{2}}} - 1}}{{\sqrt {2\pi } \rho {B_{\mathrm {rms}}}}} \tag{6a}\end{equation*}	The average fading duration
Level Crossing Rate	\begin{equation*}N(\rho ) = \sqrt {2\pi } \rho {B_{{\mathrm {rms}}}}{e^{ - {\rho ^{2}}}} \tag{6b}\end{equation*}	The average number of downward crossings of 2dB threshold per second
50% Coherence Time	\begin{equation*}{R_{\mathrm {yy}}}(m) = \frac {{\sum \limits _{n = 1}^{L - m} {\left \{{ {y(n) - {m_{y}}} }\right \}\left \{{ {y^{*}(n + m) - m_{y}^{*}} }\right \}} }}{{\left |{ {r(n)} }\right | \times \left |{ {r(n + m)} }\right |}}\tag{6c}\end{equation*}	Representative of speed of fluctuations in signal in a location
Rayleigh Parameter	\begin{equation*}{f_{\mathrm {ray}}}(r) = \frac {y}{\sigma ^{2}}\exp \left({ - \frac {y^{2}}{{2{\sigma ^{2}}}}}\right),{\mathrm{ y}} \ge 0\tag{6d}\end{equation*}	Representative of speed of fluctuations in signal in a location
Frequency Domain Features		
Energy	\begin{equation*}E = \int {D(\lambda )d\lambda } \tag{7a}\end{equation*}	The energy of Doppler spectrum
Laplacian Best Fit	\begin{equation*}D(\lambda ) = \frac {a}{{1 + b{\lambda ^{2}}}}\tag{7b}\end{equation*}	b is the bell shape fit parameter
Rms Doppler Spread	\begin{equation*}{B_{\mathrm {rms}}} = \sqrt {\frac {{\int {\lambda ^{2}D(\lambda )d\lambda } }}{{\int {D(\lambda )d\lambda } }}} \tag{7c}\end{equation*}	The second central moment of Doppler spectrum
General Statistical Features		
Mean	\begin{equation*}\frac {1}{L}\sum \limits _{n = 1}^{L} {y(n)} \tag{8a}\end{equation*}	The central value of a window
$Y_{p2p}$	\begin{equation*}{\left .{ {\left \{{ {y(n)} }\right \}} }\right |_{\max }} - {\left .{ {\left \{{ {y(n)} }\right \}} }\right |_{\min }}\tag{8b}\end{equation*}	Peak-to-peak changes in RSSI of one location
Standard Deviation	\begin{equation*}\sqrt {\frac {1}{L}\sum \limits _{n = 1}^{L} {(y(n) - \bar y)^{2}} } \tag{8c}\end{equation*}	A measure of variations about the central value
Interquartile range	\begin{equation*}\mathrm {IQR} = {Q_{3}} - {Q_{1}}\tag{8d}\end{equation*}	A measure of variability, based on dividing a data set into quartiles
Skewness	\begin{equation*}E\left[{{\left({\frac {(y(n) - \bar y)}{\sigma }}\right)^{3}}}\right]\tag{8e}\end{equation*}	A measure of the asymmetry about the mean value of data set
Kurtosis	\begin{equation*}E\left[{{\left({\frac {(y(n) - \bar y)}{\sigma }}\right)^{4}}}\right]\tag{8f}\end{equation*}	A measure of whether the data set is heavy-tailed or light-tailed relative to a normal distribution

#### 1) Crossing Rate and Duration of the Fades

**FIGURE 4.**
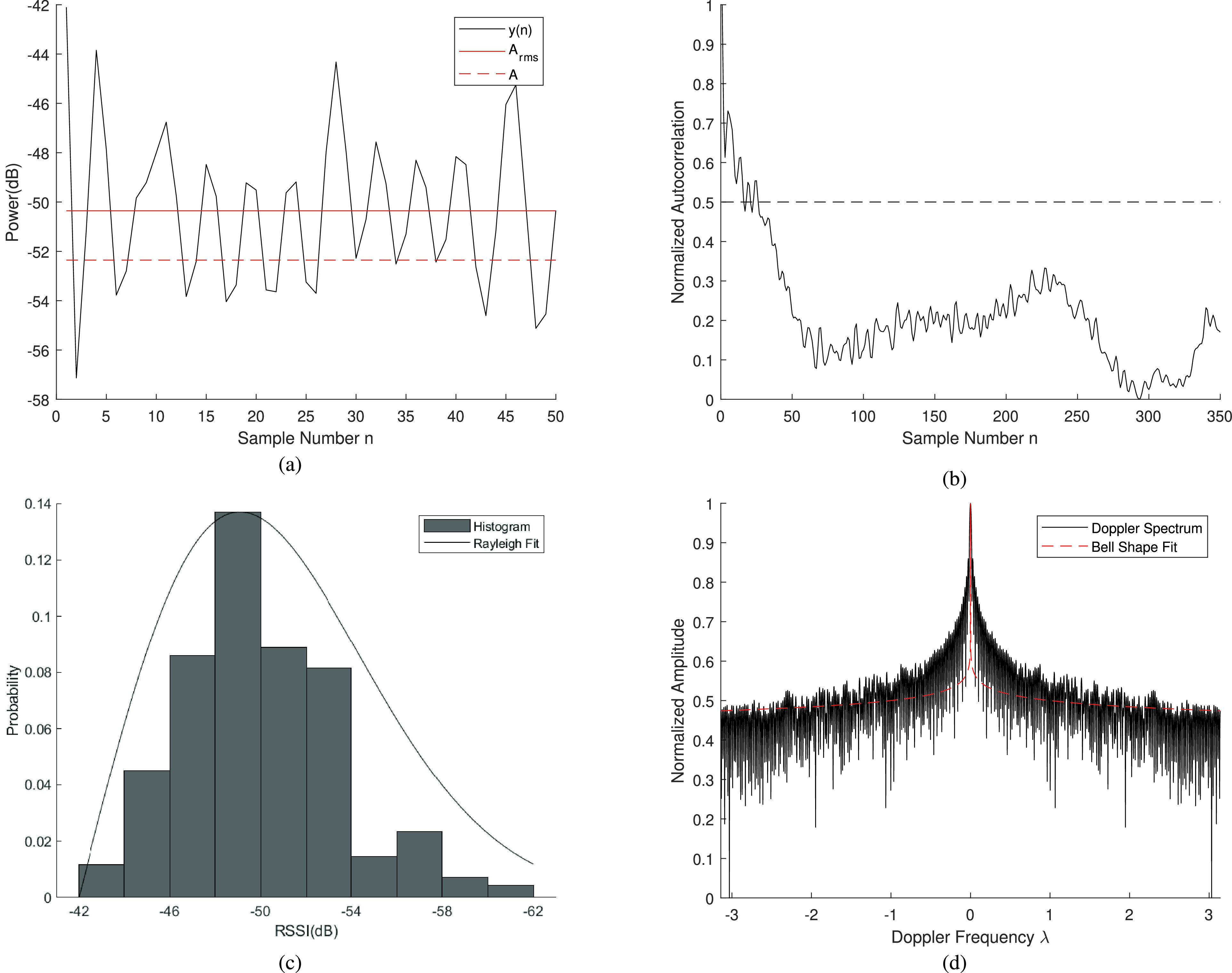
Summary of time- and frequency-domain features extracted from samples of MRAS RSSI data a) level crossing rate and fade duration and their relation to rms Doppler spectrum for a sample, b) 50% Coherence time using the autocorrelation function, c) Rayleigh fit for distribution of amplitude fluctuation, and d) Laplacian fit to doppler spectrum.

Fig. 4.a shows a sample of the fluctuations of RSSI sequences, $y(n)$, over time caused by small scale temporal fading characteristics of the channel. Two interesting features of short-range fading for RSSI measurements are the fading rate and fading durations. We can calculate the rate of fluctuation of the envelope of the RSSI caused by multipath fading with these parameters. It is well known that in Rayleigh fading channels the threshold crossing rate, $N(\rho )$, and average duration of fade, $\tau (\rho )$, are related to the rms Doppler spread $B_{\mathrm {rms}}$ (see section 3.3). Fig. 4.a shows the definition of the fade rate and the duration of the fade as well as equations relating them together on a sample of MRAS measured data. Defining the normalized crossing threshold as, $\rho =A/A_{\mathrm {rms}}$, in which $A$ and $A_{\mathrm {rms}}$ are the threshold level and rms amplitude of the RSSI, respectively, these relations are given by (6a) and (6b) [23] in the top two rows of the time-domain features in Table 2. Given a set of data in a location (Fig. 4.a) we find the fading rate and fade duration for the signal and use these values as features to train the ML algorithm.

#### 2) Coherence Time

Another feature to determine the speed of fluctuations in values of RSSI is the coherence time of the signal [23]. The coherence time is the width of the correlation function of the samples of the RSSI in a location. For L samples of RSSI defined by sequence $y(n)$, (3), the normalized autocorrelation function is given by (6c) in Table 2, in which

\begin{align*} \begin{cases} {m_{y}} = \frac {1}{L}\sum \limits _{n = 1}^{L} {y(n)} \\ r(n) = \sqrt {\frac {1}{L}\sum \limits _{n = 1}^{L} {[y(n)} - {m_{y}}{]^{2}}}. \end{cases}\end{align*}

As shown in Fig. 4.b, the value of the plot at the intersect with the 50% line is used as the coherence time. We can use the coherence time as another time-domain feature of the small scale fading in a location to train a ML algorithm for ranging.

#### 3) Shape of Fading Distribution

Multipath fading results in fluctuations of the signal amplitude because of the addition of signals with different phases arriving from multiple paths. This phase difference is caused by the signals traveling different distances along multiple arriving paths. Since the phase of the arriving signals changes rapidly, the received signal amplitude undergoes rapid fluctuation that is often modeled as a random variable with a Rayleigh distribution given by (6d) in Table 2 [23], where $\sigma _{\mathrm {ray}}$, is the standard deviation of the Rayleigh distribution function. To model these fluctuations, we can generate a histogram of the amplitude of the received signal in time and fit it to a Rayleigh distribution function. Fig. 4.c shows a sample Rayleigh fit to the MRAS data, by fitting the Rayleigh distributions to a set of MRAS data, $y(n)$, we can determine $\sigma _{\mathrm {ray}}$, the parameters defining the distribution of that data and then associate that parameter as a feature per-location for training the ML algorithm.

### C. RSSI Feature in Frequency Domain

Traditionally, RSSI of RF signals have been used in Doppler radars and for GPS signals to measure the speed to correct the estimated range for moving objects. In recent years, using RSSI signal in time and in frequency with intelligent algorithms have attracted attention in new emerging fields for big data such as gesture [26] and motion detection [18], [19]. Parameters associated with the Doppler spectrum can also be used for range estimation using ML algorithms. Doppler spectrum, $D(\lambda )$, is the magnitude square of the Fourier transform of variation of the signal in time domain For a discrete sequence, $y(n)$, using the Fast Fourier Transform (FFT), we can calculate samples of Doppler spectrum function as:

\begin{align*} \begin{cases} Y(k) = \mathrm {FFT}\left [{ {y(n)} }\right ]\\ {\left .{ {D(k) = D(\lambda )} }\right |_{\lambda = k/{T_{s}}}} = {\left |{ {Y(k)} }\right |^{2}}. \end{cases}\end{align*}

We extracted three features from the empirical Doppler spectrum obtained from a set of data at a location, $y(n)$. The middle part of Table 2 summarizes these frequency domain features. These parameters are the energy of the signal, $E$, defined by (7a) in Table 2, the RMS Doppler spread, defined by (7b), and the shape of the Doppler spectrum in indoor areas, defined by (7c). The RMS Doppler spread is the normalized second moment of the $y(n)$, reflecting the speed of motions in the environment. We used this parameter in the previous section for calculating the fade rate and duration as well. According to the IEEE 802.11 standard organization model for the RSSI, the Doppler spectrum shape follows a Laplacian distribution in indoor areas, shown in (7b) of Table 2 [22]. By normalizing amplitude to one, $a=1$. Fitting with empirical data results in a single parameter, $b$, reflects the speed of BLE RSSI fluctuations at a given location. Fig. 4.d shows a sample of the FFT and the best fit Laplacian function for the MRAS RSSI data. In this way, we extract three parameters to represent the frequency domain characteristics of each data sequence y(n), {$E$, $B_{\mathrm {rms}}$, $b$}, and use these three features along with the four time-domain features and the following six statistical features for training ML algorithms using the MRAS data.

### D. Statistical Features of RSSI

The features of the signal that we referred to so far in this section have physical meanings that we borrowed them from the multipath RF propagation literature [22]. The set of RSSI data in a location can also be treated as a mathematical sequence from which we calculate statistical features that are then fed as inputs to ML algorithms. In this study, we have included six common statistical features of the RSSI samples at a location, shown in the last six columns of Table 2, for training the ML algorithms. The two top rows of statistical features are mean and peak-to-peak changes of the RSSI sample at a location. Other traditional RSSI features are Interquartile Range (IQR), which shows the spread of RSSI. Skewness and Kurtosis, which are parameters depicting the shape of RSSI distribution.

## IV. Proximity Detection Algorithms

The objective of this study is to investigate the accuracy of classical estimation theory algorithms for Covid-19 proximity detection and compare their performance with the results obtained using ML algorithms. Classical algorithms use the empirical measurements to model the behavior of RSSI with (4) and (5), then calculate the confidence on the estimation based on parameters of these models. This approach enables faster computation in a more logically explainable manner and it also enables us to calculate the Cramer-Rao Lower Bound (CRLB) of the performance achievable by any algorithm. ML algorithms benefitting from all the spatial, time, frequency, and traditional statistical features of the RSSI shown in Table 2, solving the same problem, and providing their level of confidence on these estimates. In the remainder of this section, we describe the details of these two classes of algorithms that we have used in this study.

### A. Classical Estimation Algoritms

Classical estimation theory provides methods for modeling, estimating, and calculating the performance bounds of an estimator. In the classical estimation theory terminology, estimation of the range using the RSSI, $P_{r}$, defined by (2), is referred to as estimation of a single parameter, the range $r$, using observation of the function of the parameter, $g(r)$, in additive Gaussian noise, the shadow fading $X(\sigma )$,

\begin{equation*} O:{P_{r}} = g(r) + X(\sigma ). \tag{9a}\end{equation*}

In our problem, we have a traditional RSSI linear regression model, (4), and its alternative BLE specific model, (5) [25]:

\begin{align*} \begin{cases} {g_{1}}(r) = {P_{0}} - 10\alpha {\log _{10}}(r)\\ {g_{2}}(r) = {P_{0}} - A\cos (2\pi d/\lambda ) + 10\alpha \log (r). \end{cases} \tag{9b}\end{align*}

When we establish the model the classical estimation theory provides us with tools for systematic estimate of the range and the analysis of the accuracy of the estimation. Given an RSSI value we can estimate the distance and calculate the confidence on accuracy of that estimation in observing the social distance. In addition, classical estimation theory provides tools for calculation of variance of the estimate using CRLB on accuracy of a single measurement and optimal confidence expected from estimation using any algorithm.

#### 1) Empirical Range Estimation and Confidence

In classical estimation theory the optimal estimate of the range, $\hat {r}$, for an average RSSI measurement of a device, $P_{r}$, defined in (2) taken at a specific range, $r$, is found by solving:

\begin{equation*} \hat r = {g^{ - 1}}(O) = {g^{ - 1}}({P_{r}}). \tag{10a}\end{equation*}

For a traditional linear regressive model, (4), we have a closed form answer for the problem:

\begin{equation*} \hat r = {g}^{ - 1}({P_{r}}) = {10^{ - \frac {{P_{r} - {P_{0}}}}{10\alpha }}}. \tag{10b}\end{equation*}

For the alternate BLE specific model, (5), we find the numerical solution to

\begin{equation*} {P_{r}} = {P_{0}} - A\cos (2\pi r/\lambda ) + 10\alpha \log (\hat r). \tag{10c}\end{equation*}

If the estimated range is less than or equal to the admissible social distance of 6 ft given that the device was also within 6 ft range; or when the estimated range is more than 6 ft and device range is also more than 6 ft, we are confident that the algorithm works properly. Therefore, the confidence on the estimate of the classical algorithms for BLE RSSI measurements at a given distance $r$ is calculated from [21]:

\begin{align*} \gamma (r)\,\,=&\Pr \left \{{ {\left [{ {\hat r \le 6/{P_{r}} \le {P_{6}}} }\right ] \cap \left [{ {\hat r > 6/{P_{r}} > {P_{6}}} }\right ]} }\right \}\, \\=&1 - \frac {1}{2}\mathrm {erfc}\left ({ {\frac {{\left |{ {P_{6} - {P_{r}}} }\right |}}{\sqrt {2} \sigma }} }\right ), \tag{10d}\end{align*}

where $P_{6}=g(6)$ is the expected RSSI measured at 6 ft distance obtained from RSSI behavior model and $\sigma $ is the standard deviation of the shadow fading. For our empirical analysis of the classical estimation methods, we have used (10d) to calculate the confidence on any set of test data. We will explain these in more details in the introduction to section V and Fig. 6.

**FIGURE 5.**
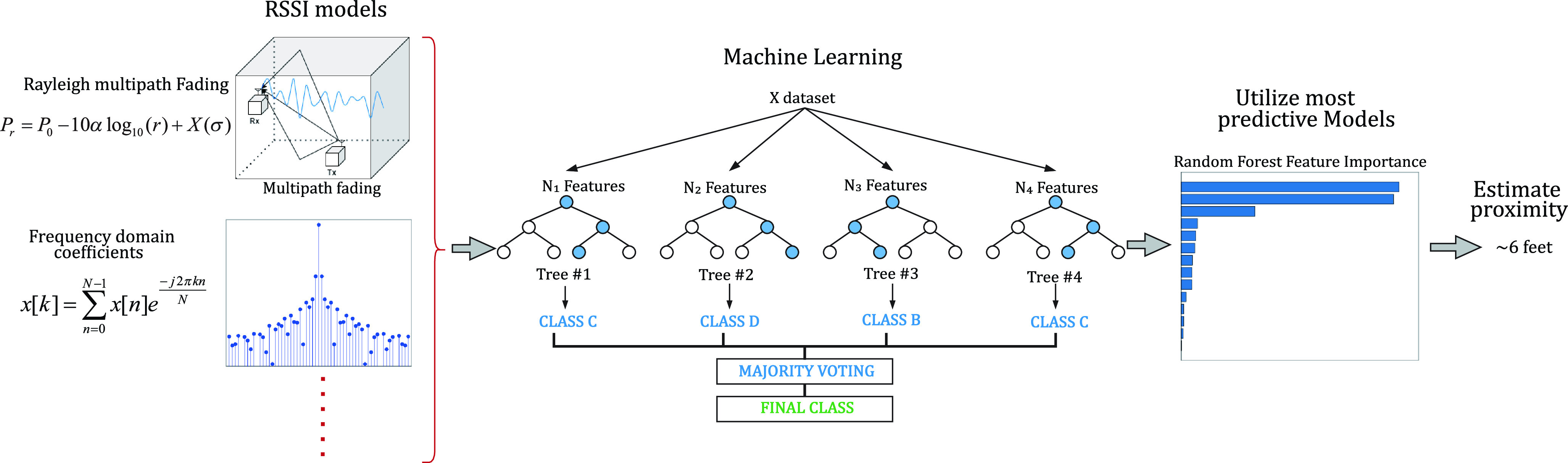
Overview of hybrid model-based ML proximity detection approach.

**FIGURE 6.**
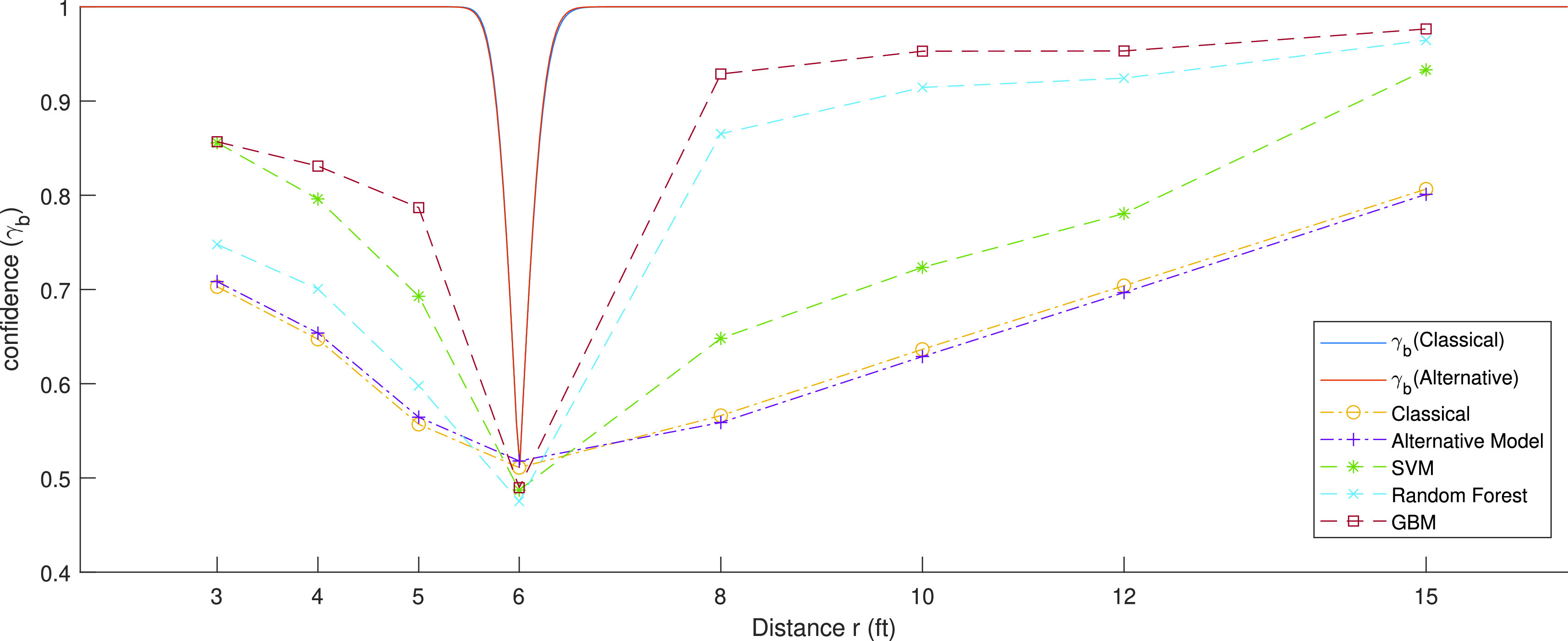
Bounds on confidence on estimation as a function of distance for MRAS RSSI database (top lines) versus performance of classical and alternative RSSI behavior modelling range estimation as well as SVM, Random Forest and GBM ML algorithms.

#### 2) Bounds on Ranging and Confidence

Another power tool from classical estimation theory is the CRLB, which is a bound on the variance of the ranging error and it is the inverse of the Fisher Information Matrix (FIM) of the dataset [21]:

\begin{equation*} {\sigma ^{2}}(r) = \mathrm {CRLB} \ge {{\mathrm {FIM}}^{ - 1}} = {\frac {{{\left [{ {g'(r)} }\right ]}^{2}}}{\sigma ^{2}}}, \tag{11a}\end{equation*}

$\sigma (r)$ is the standard deviation of the ranging error, $\sigma $, is the standard deviation of shadow fading at the location, and $g(r)$ is the function representing the model for the two models of RSSI behavior we studied. Substituting the two models given in (9b) in to (11a), we have:

\begin{align*} \begin{cases} {\sigma _{1}}(r) = \sqrt {\mathrm {CRLB}} \ge \dfrac {\ln 10}{\sqrt {N} 10}\dfrac {\sigma }{\alpha }r\\ {\sigma _{2}}(r) = \sqrt {\mathrm {CRLB}} \ge \dfrac {\sigma }{{\sqrt {N} \left({\dfrac {2\pi A}{\lambda }\sin \left({\dfrac {2\pi r}{\lambda }}\right) + \dfrac {10\alpha }{\ln 10 \cdot \alpha }}\right)}}. \end{cases} \!\!\! \\{}\tag{11b}\end{align*}

Equation (11a) provide bounds on the variance of estimate using the two RSSI behavior models at a given location. In our COVID-19 social distancing problem, we are interested in measuring our confidence in a distance estimate. The confident is the probability that the estimated range is less than or equal to the admissible social distance of 6 ft given that the device was in fact within the 6 ft range; or probability that the estimated range is more than 6 ft and device range is also more than 6 ft. If we assume that distance measurement error is a zero mean Gaussian random variable, we can model the distance estimate by

\begin{equation*} \hat r = r + \eta \left [{ {\sigma (r)} }\right ],\end{equation*}

where $\eta \left [{ {\sigma (r)} }\right ]$ is the measurement noise calculated from the CRLB of (11a). Therefore, given a distance, $r$, and assuming that the distance measurement error is a zero mean Gaussian random variable, we can calculate our confidence in making a measurement in one side of 6 ft and estimating it on the correct side from:

\begin{align*} \gamma (r)\,\,=&\Pr \left \{{ {\left [{ {\hat r \le 6/r \le 6} }\right ] \cap \left [{ {\hat r > 6/r > 6} }\right ]} }\right \} \\=&1 - \frac {1}{2}\mathrm {erfc}\left ({ {\frac {{\left |{ {6 - r} }\right |}}{\sqrt {2} \sigma (r)}} }\right ), \tag{12}\end{align*}

where $\gamma (r)$ is the bound on confidence of estimating the distance using RSSI observed at a distance $r$, and $\sigma (r)$ is the variance of estimation defined by (11a). Equations (11a) and (11b) demonstrate the bounds on estimating a location from classical models for RSSI behavior, an ideal expected confidence while (12) demonstrates the confidence on actual measurements. Equations (10d) an algorithm for calculation of confidence from the empirical data. In section V, we use these equations to calculate the bounds on the confidence of range estimation as well as the confidence of the RSSI measurement based ranging using empirical data (see Fig. 6 in that section).

### B. Machine Learning Algorithms

In classical range estimation, only the spatial characteristics of RSSI measured values are utilized without considerations its temporary characteristics in time and in frequency domain. ML algorithms can benefit from other features of the signal, providing a better estimate of the range and distance and improving the confidence of the result of estimation. In this paper we intend to compare these two approaches quantitatively. In practice the ML approach is more computationally sophisticated, and the classical approach is more analytically complex but simpler to implement. The classical approach relies on modelling, which enables faster computation in a more logically explainable manner. Moreover, classical approaches generalize better to new, previously unseen scenarios. While prior work has typically used either the classical or ML approaches, we explore combining both methods creating a third hybrid approach that uses classical models and parameters as inputs to the ML algorithms, facilitating model-based ML algorithms. In a sense, this is the best of both worlds, integrating the temporal characteristics in time and frequency as well as model parameters (see Fig. 5). Model based ML has shown to be effective in RSSI based motion and gesture detections to reduce the complexity and computational time of the algorithms [18], [19], [27], in this paper we have examined them for the proximity range estimation for COVID-19 social distancing with BLE signals.

In the remainder of this section, we review the three ML algorithms that we have considered in this study and used in our comparative performance evaluation among classical: Random Forest, Gradient Boosted Machines and Support Vector Machines.

#### 1) Random Forest

Random Forest is an ML classification algorithm that is an ensemble of $K$ classifiers $M_{i},\ldots M_{K}$, where each classifier is a decision tree created using a different sub-sample of the entire dataset [27]. The final classification is obtained by majority voting of the $K$ decision trees $M_{1}~M_{2},\ldots M_{K}$. For a new test point $ \boldsymbol {x}$, the class predicted by the Random Forest model $ \boldsymbol {M}^{K}$ using majority voting is:

\begin{equation*}{{ \boldsymbol {M}^{K}}}({ \boldsymbol {x}}) = \mathop {\arg \max }\limits _{c_{j}} \left \{{ {v_{j}| j = 1,\ldots .k} }\right \},\end{equation*}

where $v_{j}$ is the number of trees created from the different dataset sub-samples, which predict the class of $ \boldsymbol {x}$ as $c_{j}$. That is,

\begin{equation*}{v_{j}}({ \boldsymbol {x}}) = |\left \{{ {{{ \boldsymbol {M}}^{K}} = {c_{j}}|t = 1,\ldots K} }\right \}.\end{equation*}

#### 2) Gradient Boosted Machines (GBM)

We explored classification using XGBoost, a high-performance implementation of GBM also called Gradient Boosted Trees [28]. The GBM classification model is an ensemble model that uses $K$ additive functions to predict the output:

\begin{equation*}\hat y = \phi ({{ \boldsymbol {x}}_{i}}) = \sum \limits _{k = 1}^{K} f_{k}({{ \boldsymbol {x}}_{i}}),\quad {f_{k}} \in F,\end{equation*}

where $F$ is the space of regression trees created from different subsets of the input dataset. To learn the set of functions utilized by the model, the following regularized objective is minimized

\begin{equation*}L(\phi ) = \sum \limits _{i} {l(} \hat y,{y_{i}}) + \sum \limits _{k} {\Omega (} {f_{k}}),\end{equation*}

where $l$ is a differentiable convex loss function, which measures the difference between the prediction $\hat {y}$ and target $y_{i}$, and

\begin{equation*}\Omega (f)\,\,= \gamma {T} + \frac {1}{2}\lambda || \boldsymbol {w}_{\mathrm {score}}|{|^{2}},\end{equation*}

in which $T$ is the number of leaves in the tree, $\gamma $ and $\lambda $ are regularization parameters, and $ \boldsymbol {w}_{\mathrm {score}}$ is the score of corresponding leaves.

#### 3) Support Vector Machines (SVM)

SVM is a ML classification algorithm that tries to discover a hyperplane that maximizes the margin between the target classes in feature space [29] and it is based on the theory of maximum linear discriminants. For two classes to be classified, SVM finds peripheral data points in each class that are closest to the other class (called support vectors). For a dataset $D$ with $n$ points $ \boldsymbol {x}_{i}$ in a $d$-dimensional space, a hyperplane function $h( \boldsymbol {x})$ can be defined as

\begin{equation*}h({ \boldsymbol {x}}) = { \boldsymbol {w}^{\mathrm {T}}}{ \boldsymbol {x}} + b = {w_{1}}{x_{1}} + {w_{2}}{x_{2}} + \ldots + {w_{d}}{x_{d}} + b,\end{equation*}

where $ \boldsymbol {w}$ is the weight vector. Overall, $n$ points, the margin of the linear classifier can be defined as the minimum distance of a point from the separating hyperplane given as:

\begin{equation*}{\delta ^{*}} = \min \limits _{{{ \boldsymbol {x}}_{i}}} \left \{{ {\frac {{y_{i}({{ \boldsymbol {w}}^{T}}{{ \boldsymbol {x}}_{i}} + b)}}{{||{ \boldsymbol {w}}||}}} }\right \}.\end{equation*}

The SVM classifier finds the optimal hyperplane dividing the two classes by solving the minimization problem with objective function:

\begin{equation*}\min \limits _{{{ \boldsymbol {w}}_{i}}b} \left \{{ {\frac {{||{ \boldsymbol {w}}|{|^{2}}}}{2}} }\right \},\end{equation*}

with linear constraints:

\begin{equation*}h({ \boldsymbol {x}}) = {y_{i}}({ \boldsymbol {w}^{T}}{ \boldsymbol {x}_{i}} + b) \ge 1,\quad \forall {{ \boldsymbol {x}}_{i}} \in {D}.\end{equation*}

Then, the class of a new point $ \boldsymbol {z}$, is predicted as:

\begin{equation*}\hat y = {\mathrm {sign}}(h({ \boldsymbol {z}})) = {\mathrm {sign}}({ \boldsymbol {w}^{\mathrm {T}}}{ \boldsymbol {z}} + b).\end{equation*}

#### 4) Confidence Calculation

To compare the performance of ML Classifiers with that of the classical estimation theory, it is necessary to calculate the confidence of the classifications generated by the SVM, Random Forest, and GBM classifiers. To calculate this confidence, we split the training data into 8 groups based on the distance between the transmitter and the receiver. Then we calculated, the confidence at each distance from:

\begin{equation*} \gamma (r)\,\,= \Pr \left \{{ {\left [{ {\hat r \le 6/r \le 6} }\right ] \cap \left [{ {\hat r > 6/r > 6} }\right ]} }\right \}, \tag{13}\end{equation*}

which represents the probability of estimating the distance to be at the correct side of 6 ft social distance barrier. These results from ML algorithms are comparable with the results obtained from (13) for the two classical approaches from the traditional linear regression and the BLE specific models for RSSI behavior. These experimental results are then compared with the bounds on confidence in (12) obtained from calculation of the CRLB.

## V. Performance of Ranging With BLE Signals

In this section we present the results of applying the algorithms described in section IV, using the BLE RSSI features described in section III, on the PACT MRAS dataset that was described in section II. The basic performance criterion we use is the confidence on correctly estimating the social distance of 6 ft between smartphone users. That is, the probability of correctly detecting the distance of a device relative to the 6 ft threshold using RSSI measurements. We begin by calculating bounds on confidence of RSSI based ranging (section IV.A2), then we present the results of classical estimation theory ranging (section IV.A1), and finally results from ML algorithms (section IV.B4).

As the first step, the training data is used for calculating parameters of the traditional RSSI behavior models using the LS algorithm. The model parameters are then used to calculate the CRLB and then the bounds on the confidence. Then, the test data and model parameters are used for calculating results from the test data to determine the confidence on classical methods with the two RSSI behavior models. Finally, we trained the three ML algorithms using the training data and found the confidence on estimation for the test data to compare with results of classical methods as well as bounds on the performance. We refer to the results of calculating the bounds on the test data using spatial RSSI behavior models as classical performance evaluation and we present those results first. All the test scenarios shown in Table 1 are for the Line-Of-Sight (LOS) propagation condition without any object obstructing the LOS path between the transmitter and the receiver and the maximum distance is 15 feet (Fig. 1.b). We begin by presenting the results for our traditional RSSI behavior model described by (4). We have 40 sets of data for five scenarios in eight distances. We utilized 75% of the data to estimate the parameters of the RSSI behavior model with LS algorithm. The model is trained for LS estimation with the RSSI averaged at each distance shown in the measurement scenario of Fig. 1.b. The three parameters of the traditional RSSI regressive model, power at the reference point, distance-power gradient, and standard deviation of shadow fading, were $P_{0} = -54.94\mathrm {~dBm}$, $\alpha = 1.74$ and $\sigma = 3.78\mathrm {~dB}$, respectively. We repeat the same procedure on training data to calculate the five parameters of the alternative BLE specific model. These parameters, power at the reference point, scale factor, spatial wavelength, distance-power gradient, and the standard deviation of shadow fading, were calculated as $P_{0} = -55.88\mathrm {~dBm}$, $A = -0.93$, $\lambda = 11.31$, $\alpha =1.51$, and $\sigma =3.78\mathrm {~dB}$, respectively.

### A. Effects of Distance on Confidence

With these parameters of the RSSI behavior models estimated using LS estimation, we calculated the bound on standard deviation of the range measurement error using the CRLB for the two, classical and BLE specific, models from (15a) and (15b), respectively. These bound are then applied to (16) to determine the bounds on confidence on the estimate as a function of distance, $\gamma (r)$, for the two RSSI behavior models. The solid line on top of Fig. 6 shows the plot of bounds on confidence on estimation as a function of range, $\gamma (r)$, for the two path loss models. As shown in this figure, although alternative model was providing a better estimate of the RSSI values (Fig. 3), the confidence for estimating the range from either of the models with the CRLB, remains almost the same. Alternative model provided a slightly better performance than the classical linear regression model in Fig. 3 because it fits better to BLE data before 3.5 m. With the BLE RSSI, for less than 1.5 m, we are almost 100% confident in the estimate enabling us to overrule the social distance range of 6 ft and for distances of more than 2.5 m we have the same confidence that we are observing the social distance rule. Since models are based on zero mean Gaussian modeling of the noise, at the exact distance of 6 ft the best algorithms can only detect the range with 50% confidence. The bounds on confidence, $\gamma (r)$, plots in Fig. 6 show us best expected confidence that we obtain from RSSI measurements in our test dataset.

As the next step, we examined the performance of classical estimation models from solving (12c) to estimate the distance from the test data. In this part we use the average RSSI’s for each distance, $r$, in the remaining 25% of database to solve (12c) to find our estimate of the distance, $\hat {r}$, with each of the RSSI behavior models. Then we empirically calculated the confidence from (17) for any specific data set. The bottom lines of Fig. 6 show the performance of the range estimation with traditional and BLE specific alternative models obtained from empirical studies. Performance of the classical estimation algorithms follow the V-shape of the bounds on the performance, but the quality of estimate is substantially lower than the bounds. This encouraged us to examine ML algorithms to improve the performance. The dashed-lines in Fig. 6 show the results of applying the three different ML algorithms examined in this paper (section IV.B). The three ML algorithms, SVM, Random Forest, and GBM will improve the performance over the classical methods, when the social distance of the device is 6 ft or larger. However, on or in the proximity of 6 ft classical models perform slightly better.

### B. Effects of Environment and User Behavior

In the last section we presented the results of effects of range on confidence of estimate with classical and ML algorithms and we compared that with performance bounds that are achievable as calculated using CRLB. Now that we established the framework for the analysis against the bounds and trained our algorithms with the training database, it is possible to explore the relationship between scenarios of operation and the behavior of the user on the expected performance. In section II, Table 1, we partitioned the MRAS database into five different scenarios for the test. We classified the top two scenarios in the Table 1, describing the area size and relative location in the area, as scenarios related to the effects of the environment, and the last two scenarios, describe the location in which the smartphone is carried and the pose of the tester, as scenarios describing the user behavior. In this way, we divide the scenarios into, the environment and user behavior, and analyzed them separately for each of the seven different devices, in the middle column of the Table 1. To make the comparison more focused and clearer, we first compare the performance of classical RSSI linear regression model with that of the GBM ML algorithm. As shown in Fig. 6, results of confidence analysis with traditional RSSI model and the BLE specific model are very close. The average of confidence over all distances for the classical method using the traditional linear regression in this figure is 69.60% and the average confidence for the BLE specific model is 69.55%. As the alternative model has almost the same average confidence as the classical model and the traditional algorithm offers an easier and more physically explainable method for estimation, we only compared the classical model with the best ML algorithms. The GBM classifier has highest average confidence of 89.58% for the entire dataset, the average for Random Forest is 84.88%, and SVM has an average of 73.20% in confidence. All three ML algorithms benefitted from all thirteen features of the RSSI and performed better than the classical models. Overall, GBM achieves the best results. Therefore, the comparison of GBM and traditional RSSI behavior modeling for our specific problem illustrates the best performance that the classical and ML algorithms can achieve with our existing dataset.

We began our analyses of the effects of various parameters on the performance by looking at the results in different environments. Table 3 shows the confidence in different environmental settings for all tested smartphones with the classical RSSI model and GBM. The MRAS dataset utilized in this study was collected by multiple currently cohabiting testers and in multiple random scenarios to create a comprehensive dataset. The tests were conducted in various residential buildings or public spaces representing a variety of architecture and five different space sizes. The testers had to strictly obey social distancing guidelines if the test was conducted in publicly accessible locations [1]. The best indoor results are obtained in medium rooms, near the walls, in congested areas, by iPhone XS (up to 87.80% for classical and up to 95.77% with GBM) and in outside, the center of the open areas, by iPhone 8 (86.09% for classical and 97.35% for GBM). The worst indoor result for the classical is obtained in medium rooms, the center of room, in open areas, by iPhone XS Max (50.81%). The worst indoor result for GBM is obtained in medium rooms, near walls, in congested areas, and by iPhone 11 (75.88%). The worst outdoor result in open areas for classical is obtained by iPhone 7 Plus (64.30%). The worst outdoor result in open areas for GBM is obtained by iPhone XS(84.41%). The average confidence for indoor environment is 70.20% for classical, which is about 4.83% less than that of outdoor (75.03%). The average confidence for indoor environment is 87.37% for GBM which is 2.24% less than that of outdoor (89.61). The average confidences are 61.03% and 82.74% in small rooms, 70.05% and 86.38% in medium rooms, 71.51% and 89.30% in large rooms, 75.08% and 89.68% in hallways for classical and GBM respectively. The average confidence increases with the room size. On the average, GBM shows 17% improvement on confidence over results achieved by the simple classical regressive model. Table 4 compares the confidence of estimation using classical estimation approaches with using GBM ML algorithm for different user behaviors and with different devices. The best results are obtained under the scenario that both testers are standing and holding their phones in their front pants pocket, and by iPhone 8 (86.09% for classical and 97.35% for GBM). The worst result for GBM is obtained in the scenario that both testers are sitting and holding their phones in hand, by iPhone 11 (75.88%). The worst result for classical is obtained in the scenario that Tester1 is standing, Tester2 is sitting and both testers are holding their phones in their front pants pocket, by iPhone XS Max (50.81%). The average confidences are 73.60% (classical) and 88.77% (GBM) if both testers are standing, 61.37% (classical) and 79.22% (GBM) if both testers are sitting, 66.13% (classical) and 86.53% (GBM) if the Tester1 is standing and Tester2 is sitting, 76.76% (classical) and 89.66% (GBM) if Tester1 is sitting and Tester2 is standing.

**TABLE 3:** Effect of Environment on Confidence

Device as Receiver	Environment		Confidence (%)	Confidence (%)
	Room Size	Location of Tester	Classical RSSI Model	GBM
iPhone 11	Small Room	Near Wall Open	60.01	81.77
	Medium Room	Near Wall Congested	81.46	92.30
		Near Wall Open	61.88	75.88
		Center Open	51.68	78.69
	Large Room	Near Wall Open	64.11	84.76
		Center Open	78.89	93.43
	Outside	Center Open	74.69	84.49
iPhone XR	Medium Room	Near Wall Congested	66.71	89.35
		Near Wall Open	75.37	89.80
		Center Open	74.65	88.33
	Large Room	Near Wall Congested	84.73	94.96
		Center Open	69.08	88.39
	Hallway	Near Wall Open	66.75	87.13
		Center Open	82.85	87.46
iPhone 8	Small Room	Near Wall Congested	62.04	83.71
	Large Room	Center Open	56.55	94.02
	Hallway	Center Open	75.63	94.44
	Outside	Center Open	86.09	97.35
iPhone 7	Medium Room	Center Open	75.15	88.76
	Large Room	Near Wall Open	81.83	89.20
	Outside	Center Open	64.76	89.15
iPhone XS	Medium Room	Center Open	87.80	95.77
		Center Congested	67.22	80.91
	Outside	Center Open	70.69	84.41
iPhone 8 Plus	Medium Room	Center Open	81.85	80.02
	Large Room	Center Open	60.82	77.52
iPhone XS Max	Medium Room	Center Open	50.81	90.39
iPhone X	Medium Room	Center Congested	66.03	86.34
iPhone 7 Plus	Outside	Center Open	64.30	86.99
iPhone 6	Large Room	Center Open	76.05	92.08

**TABLE 4:** Effect of User Behavior (Tester’s Pose and Location of Phone) on Confidence

Device as Receiver	Pose		Phone on-body Location		Confidence (%)	Confidence (%)
	Tester1	Tester2	Tester1	Tester2	Classical RSSI Model	GBM
iPhone 11	Standing	Standing	In Hand	In Hand	78.08	88.40
				Front Pants Pocket	60.07	81.77
		Sitting	In Hand	In Hand	67.89	87.27
				Front Pants Pocket	56.91	81.61
	Sitting	Sitting	In Hand	In Hand	61.88	75.88
			Shirt Pocket	Shirt Pocket	60.85	82.56
iPhone XR	Standing	Standing	In Hand	In Hand	75.37	89.80
				Front Pants Pocket	74.51	91.49
			Shirt Pocket	Front Pants Pocket	75.18	87.37
			Front Pants Pocket	In Hand	84.74	94.96
		Sitting	In Purse	Front Pants Pocket	60.76	88.81
			In Hand	Rear Pants Pocket	76.05	81.98
				In Hand	61.71	87.78
				Front Pants Pocket	66.75	87.13
				In Purse	65.28	81.79
			Front Pants Pocket	In Hand	82.85	87.46
	Sitting	Standing	Shirt Pocket	Front Pants Pocket	78.37	90.55
iPhone 8	Standing	Standing	In Hand	In Hand	62.04	83.71
				Front Pants Pocket	75.63	94.44
			Front Pants Pocket	Front Pants Pocket	86.09	97.35
		Sitting	In Hand	In Hand	56.55	94.02
iPhone 7	Standing	Standing	In Hand	Front Pants Pocket	81.83	89.20
		Sitting	In Hand	In Hand	67.91	88.30
			Front Pants Pocket	In Hand	61.61	89.99
	Sitting	Standing	In Hand	In Hand	75.15	88.76
iPhone XS	Standing	Standing	In Hand	In Hand	79.34	90.09
			In Purse	Front Pants Pocket	67.22	80.91
iPhone 8 Plus	Standing	Sitting	In Hand	In Hand	80.02	81.85
			Shirt Pockets	In Purse	60.82	77.52
iPhone XS Max	Standing	Sitting	Front Pants Pocket	Front Pants Pocket	50.81	90.39
iPhone X	Standing	Standing	Front Pants Pocket	Front Pants Pocket	66.03	86.34
iPhone 7 Plus	Standing	Standing	In Hand	In Hand	64.30	86.99
iPhone 6	Standing	Sitting	In Hand	In Hand	76.05	92.08

### C. Effects of Number of Features in Performance of Machine Learning Algorithms

**FIGURE 7.**
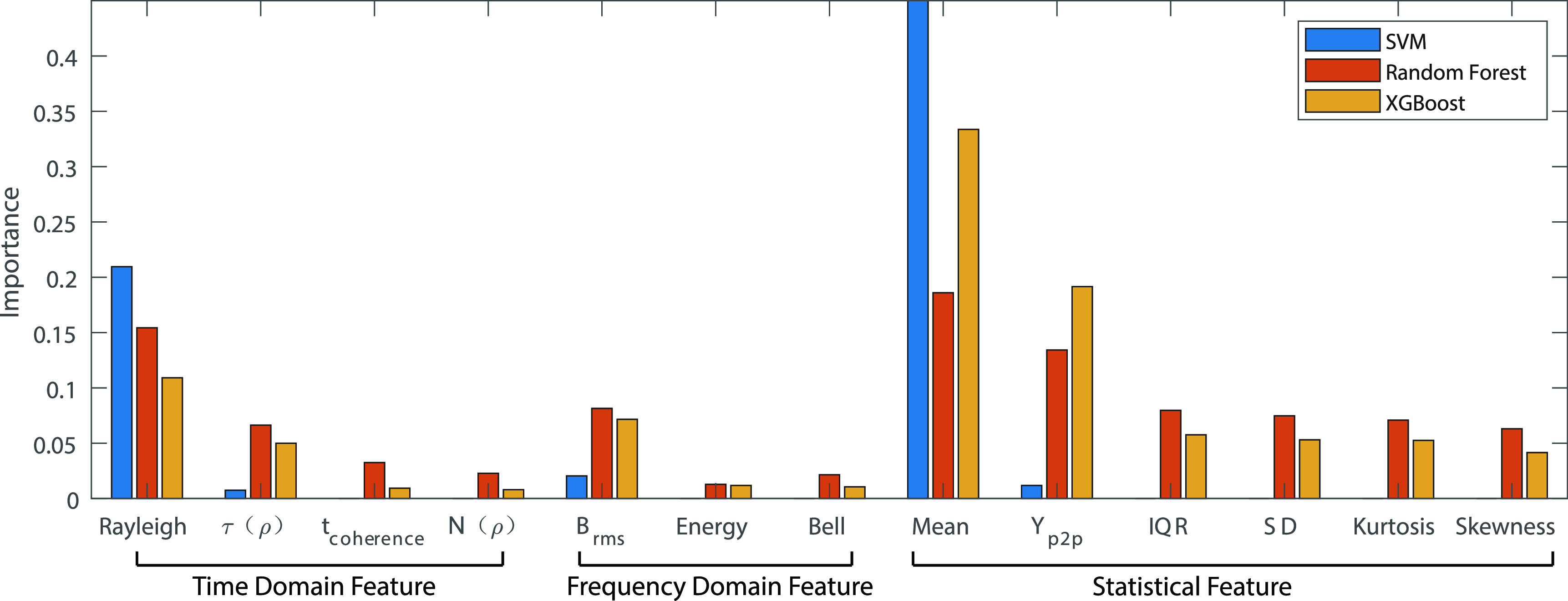
Bounds on confidence on estimation as a function of distance for MRAS RSSI database (top lines) versus performance of classical and alternative RSSI behavior modelling range estimation as well as SVM, Random Forest and GBM ML algorithms.

**FIGURE 8.**
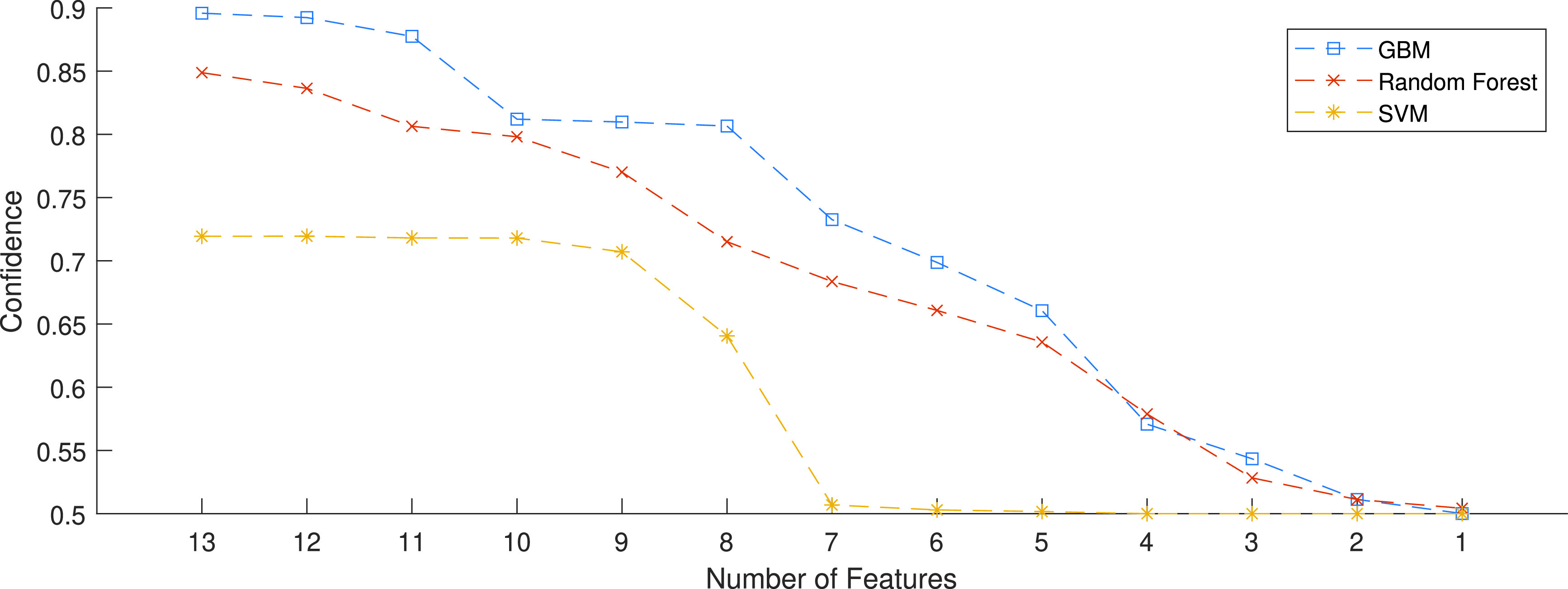
Relation between confidence and number features for SVM, Random Forest, and GBM ML algorithms as the most important of thirteen features are eliminated from training the algorithms.

We studied three Machine Learning algorithm, SVM, Random Forest, and GBM. For comparative performance evaluation of these algorithms, we trained the algorithms using the 13 features shown in Table 2, classified into three sub-groups: time-, frequency-domain, and statistical. The ML algorithms, in addition to confidence, produce measures of importance of the features. Fig. 7 shows the feature importance for the ML classifiers that we studied. As shown in the figure, the average RSSI is the most effective feature. This is the feature that we also used for classical estimation modelling. Rayleigh parameter and $Y_{P2P}$ reflecting variations in the RSSI have shown to contribute significantly. Since the MRAS dataset is collected in static environment and the Doppler Spectrum is related to the speed of moving antenna and moving object between antennas, the Frequency domain features have shown less contribution to the classification compared with the other two groups. Another traditional approach in ML is to analyze the direct effect of feature on the performance criteria, which is the confidence in the decision regarding the 6 ft threshold necessary to observe social distance. To implement this procedure, we sort the features for any of the algorithms according to their importance and re-evaluate performance while dropping them one after another. The intuition here is to demonstrate the importance of each feature on performance. Fig. 8 shows the result of gradual removal of features, each time we remove the single feature with the highest importance. The confidence of distance estimation using SVM drops significantly after the first three features are removed, demonstrating that the first three features dominate its performance. The performance of the Random Forest classifier drops gradually up to the removal of the first five features. For this algorithm, there is no sharp drop in performance, demonstrating that more features contribute to the model’s performance. There is no dominating contribution by certain features. The accuracy of the GBM classifier increases with the number of features and follows a similar gradual performance degradation pattern as Random Forest. As both methods are tree-based ensemble ML methods, the similarity in their performance is expected.

## VI. Conclusion

The risk of COVID-19 transmission increases if an uninfected person is less than 6 ft from an infected person for longer than 15 minutes (also called Too Close for Too Long (TCTL)). In this paper we have presented research, development, and comparative analysis of classical estimation theory methods, which enables faster computation in a more logically explainable manner and novel hybrid model-based ML approaches for proximity distance estimation using the RSSI information radiated from the broadcast channels of the BLE. Our results based on analyses of the Mitre Range Angle Structured (MRAS) PACT dataset in five different environments, with five different location for the smartphone, and eight different smartphones. Our analyses methodology provided a framework for the empirical analysis of the estimation confidence when applying both classical estimation theory and ML algorithms for solving the social distance estimation problem with BLE RSSI. We derived bounds on the confidence on estimation using RSSI of BLE as a function of distance. Then, we compared the performance of classical estimation theory ranging algorithms with that of the ML algorithms against the bound and we analyzed the effects of the environment and user behavior on the performance of the algorithms. The classical estimation theory algorithms using two models for RSSI spatial behavior were compared with three different ML algorithms (Random Forest, GBM and SVM) benefiting from thirteen features of the RSSI. Classical algorithms showed an average confidence of 69.60% in correctly estimating the social distance threshold of 6 ft. The GBM ML algorithm demonstrated that using the thirteen feature it can increase the confidence in the estimation of this social distance using BLE RSSI with an average confidence of 89.58%, which was 19.98% higher than the average confidence achieved using the classical approach.
